# Supplementary material for: Host relatedness and landscape connectivity shape pathogen spread in the puma, a large secretive carnivore
Source: Commun Biol. 2021 Jan 4;4:12. doi: 10.1038/s42003-020-01548-2 (PMC7782801; doi:10.1038/s42003-020-01548-2)
Supplement: Supplementary file 2 — Supplementary Information [file 42003_2020_1548_MOESM2_ESM.pdf]

Supplementary materials

Table S1. Summary of puma samples used in this study, pathogen data and population level host genetic diversity estimates and study area size.

Population	#Puma sampled	% qPCR§ tested±	FIV_{pco} prevalence (%)	% sequencedψ	% with host ddRADseq data	Host genetic diversity*	Geographic size of areas sampled (km^2)
**Unbounded**	103	93	41	70	78	1.93	11889
**WUI**	110	65	59	68	80	1.89	11958

§: Quantitative polymerase chain reaction. ± Some individuals were not tested due to insufficient blood volume. ψ: Both full length *pol*, *ORFA* and *env* genes sequenced.*: allelic richness from (1).

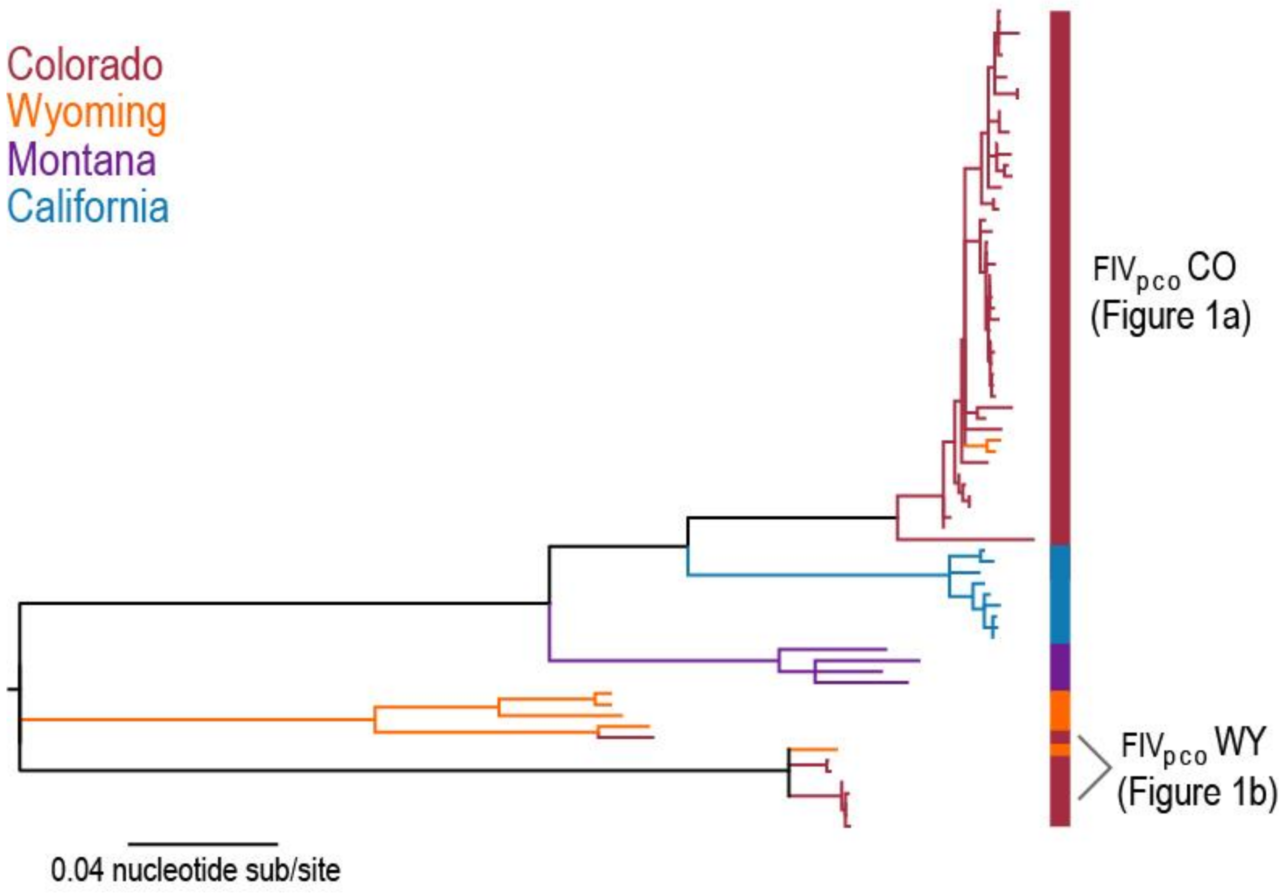

Fig. S1. Maximum likelihood tree illustrating the phylogenetic context of both subtypes found in our Colorado population compared to isolates in puma across the western U.S (GenBank accession numbers EF455603 - EF455612, KF906175 - KF906194 and MN563193 - MN563239).

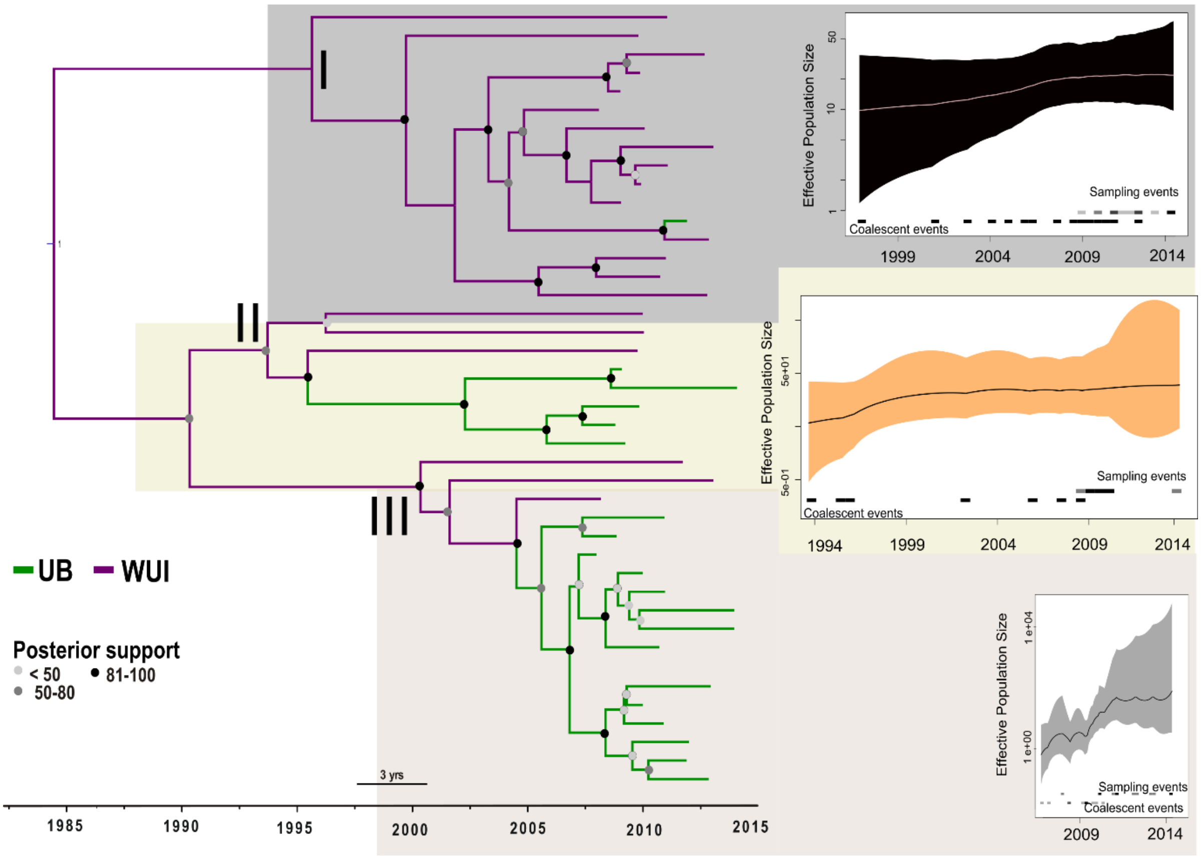

Fig. S2. Estimates of effective population size through time for each significantly different FIV_{pco} CO lineage (lineage I, II & 3, p = 0.05) based on structure analysis of our Bayesian time-scaled phylogeny (see *Methods* in the main text). The colour of the virus symbols and 95% high posterior density (HPD) intervals reflect each lineage. Edges are coloured based on the puma population the isolate was sampled from (UB: unbounded, WUI: wildland urban interface). Virus branch colours are based on population assignment posterior values from our FIV_{pco} subtype CO discrete trait analysis (see *Methods: viral phylogenetics*). Sampling events refer to dates when samples were collected from puma and coalescent events are estimated mean dates when branches coalesced.

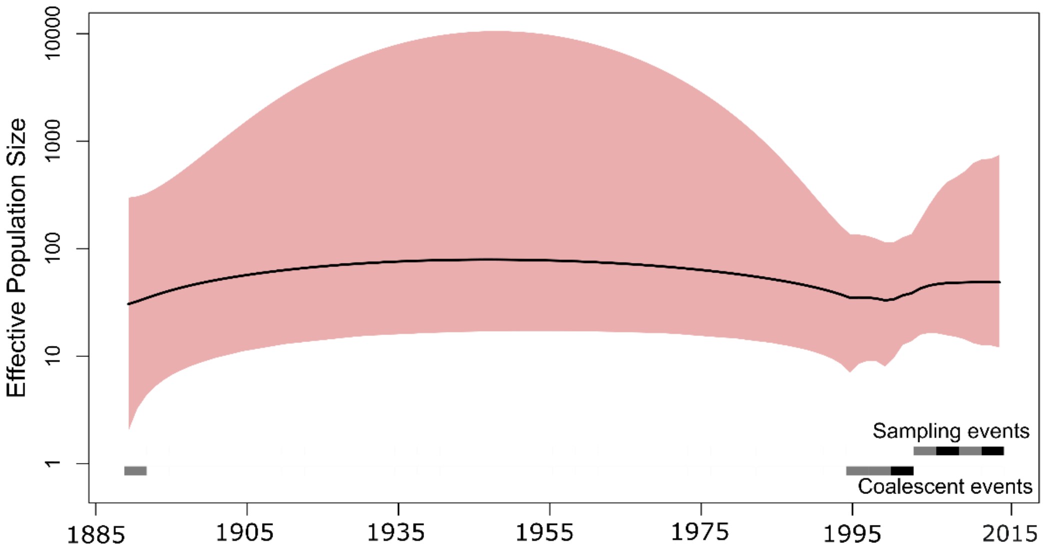

Fig. S3. Estimates of effective population size through time for FIV_{pco} WY (95% high posterior density interval in red). Sampling events refer to dates when samples were collected from puma and coalescent events are estimated mean dates when branches coalesced.

Table S2. Subtype evolutionary rates.

**Subtype**	**Evolutionary rate** (substitutions per site/yr)	**95% HPD***
FIV_{pco} WY	$1.04e^{-4}$	$2.64e^{-5}$, $2.30e^{-4}$
FIV_{pco} CO	$4.25e^{-4}$	$2.48e^{-4}$, $6.04e^{-4}$

* highest posterior density.

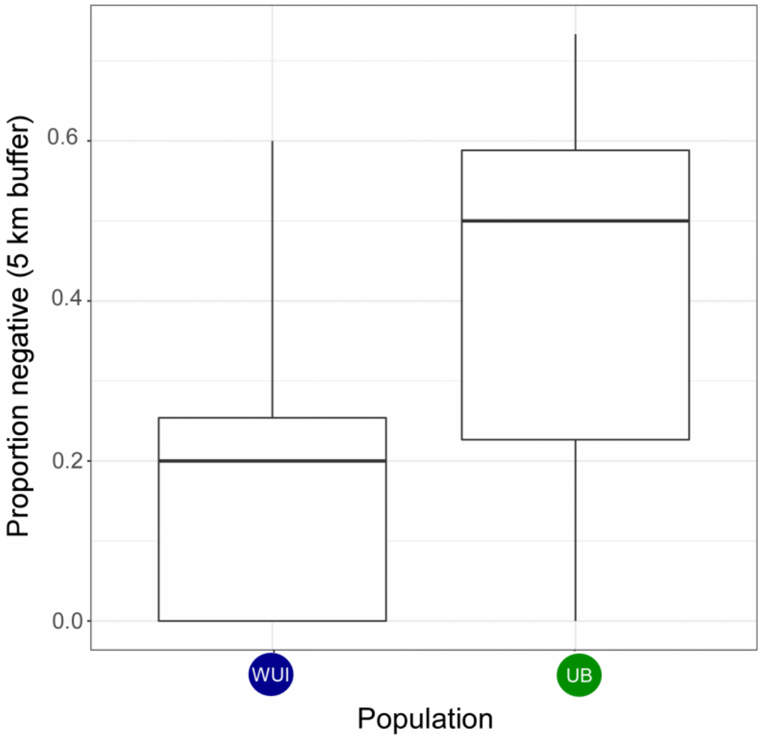

Fig. S4. Boxplot showing the proportion of individuals FIV_{pco} negative in a 5 km buffer around a positive individual in each population (WUI: wildland urban interface, UB: unbounded).

Table S3. Landscape variables used for landscape genomic analyses, source of the environmental data, and ecological justification for each variable (1)

Category	Variable	Code	Description	Data source and resolution	Calculation	Ecological justification
Distance	Isolation by Euclidean distance (null model)	Geographic proximity	Euclidean distance between individuals	National Elevation Dataset (lta.cr.usgs.gov/ned) National Map Tool (viewer.nationalmap.gov), 30 meter	Geospatial Modelling Environment (spatialecology.com/gme), ArcGIS 3D Analyst, Circuitscape	Null model of isolation by straight-line distance over variable topography (3).
Land cover	Land cover: forested, open-natural, and developed	Land cover	Multiple land cover categories collapsed into 3 costs of movement: forested (lowest), open natural areas (medium), and developed (highest)	National Land Cover Database (mrlc.gov/nlcd2011.php; Homer et al. 2011), 30 meter	ArcGIS Spatial Analyst	Forested habitats provide the most cover for hunting and dispersal, open natural areas are intermediate, and developed areas are the least suitable habitat for dispersal (4, 5).
	Percent impervious surface	Impervious	Percentage of impervious surface	National Land Cover Database (mrlc.gov/nlcd2011.php; Homer et al. 2011), 30 meter	ArcGIS Spatial Analyst	Human development results in increased noise, lights, and hunter access, limiting dispersal (6–8)
	Road corridors	Roads	Roads, with 50 meter buffers on each side	Colorado Department of Transportation (dtdapps.coloradodot.info/otis), 30 meter	ArcGIS Analysis Tools, Spatial Analyst	Roads increase mortality, noise, lights, and hunter access, limiting dispersal (6, 8, 9).
	River and stream riparian corridors	Riparian	River and stream riparian corridors, with 50 meter buffers on each side	National Hydrography Dataset (nhd.usgs.gov), 30 meter	ArcGIS Analysis Tools, Spatial Analyst	River and stream riparian corridors provide vegetative and topographical cover for dispersal, as well as water sources attracting prey species (10, 11)
Vegetation	Percent tree canopy cover	Tree cover	Percentage of tree canopy cover	National Land Cover Database (mrlc.gov/nlcd2011.php; Homer et al. 2011), 30 meter	ArcGIS Spatial Analyst	Low tree canopy limits cover for ambush predation and concealment, restricting dispersal (12–14).
	Enhanced vegetation index	Veg. density	Density of vegetation calculated from chlorophyll reflectance in visual and near-infrared spectra	Moderate Resolution Imaging Spectroradiometer (modis.gsfc.nasa.gov), 250 meter	ArcGIS Spatial Analyst	Low vegetation density limits cover for ambush predation and concealment, restricting dispersal (11, 13, 14).
Climate	Minimum temperature of the coldest month	Min. temp.	Mean annual minimum temperature of the coldest month (°C) calculated from 1970-2000 weather station data, interpolated between stations	Global Climate Data (worldclim.org/bioclim; Hijmans et al. 2005), 1 kilometer	ArcGIS Spatial Analyst	Lowest minimum temperatures, found at high elevation mountain ridgelines restrict hunting, breeding, and dispersal (15).
	Mean annual precipitation	Ann. precip.	Mean annual precipitation accumulation (mm) calculated from 1970-2000 weather station data, interpolated between stations	Global Climate Data (worldclim.org/bioclim; Hijmans et al. 2005), 1 kilometer	ArcGIS Spatial Analyst	Dry habitats with low precipitation accumulation limit prey species for hunting and vegetative cover, restricting dispersal (12, 16).
Topography	Topographic roughness	Topo. rough.	Topographic complexity based on variance in elevation within a moving window	National Elevation Dataset (lta.cr.usgs.gov/ned) National Map Tool (viewer.nationalmap.gov), 30 meter	Geomorphometric and Gradient Metric Toolbox (Cushman et al. 2010), ArcGIS Spatial Analyst	Steep, topographically-complex canyons and mountain slopes provide cover for hunting and dispersal (10, 15).
	Elevation	Elevation	Elevation calculated from digital elevation models.	National Elevation Dataset (lta.cr.usgs.gov/ned) National Map Tool (viewer.nationalmap.gov), 30 meter	ArcGIS Spatial Analyst	Higher elevation, forested habitats are better for hunting, breeding, and dispersal than lower elevation, prairie and high desert habitats.

Table S4. Results from resistance/conductance optimization using the ResistanceGA approach (2) from the unbounded (UB) region

**Surface**	**AICc**	**k**	**LL**
Null	-1083.64	1	542.9335
Spatial proximity	-1081.32	2	543.0139
Streams	-1076.65	4	543.6566
Forests	-1076.48	4	543.5737
Roads	-1076.23	4	543.4485
Savannas	-1075.89	4	543.2777
Grasslands	-1075.74	4	543.2055
Topography	-1075.6	4	543.1337
Impervious surfaces	-1075.53	4	543.096
Croplands	-1075.49	4	543.077
Canopy cover	-1075.42	4	543.0427
Temperature	-1075.42	4	543.0416
EVI	-1075.38	4	543.0211
Precipitation	-1075.38	4	543.021
Urban land cover	-1075.37	4	543.0159
Elevation	-1075.36	4	543.0139

AICc: Corrected Akaike Information Criterion, k: multiplier for the penalty term for complexity, LL: Log likelihood.

Table S5. Results from resistance/conductance optimization using the ResistanceGA approach (2) from the WUI region

**Surface**	**AICc**	**k**	**LL**
Spatial proximity	-1083.804992	2	544.2358
Canopy cover	-1082.134786	4	546.3174
Urban land cover	-1081.856408	4	546.0282
Streams	-1081.107197	4	545.8036
Elevation	-1081.105302	4	545.8027
Temperature	-1081.073311	4	545.7867
Precipitation	-1080.546697	4	545.5233
EVI	-1079.927086	4	545.2135
Grasslands	-1079.835527	4	545.1678
Savannas	-1079.391561	4	544.9458
Forests	-1079.172723	4	544.8364
Impervious surfaces	-1078.823596	4	544.6618
Croplands	-1078.793611	4	544.5854
Topography	-1078.670753	4	544.3783
Roads	-1078.25668	4	544.2358
Null	-1077.971663	1	539.954

AICc: Corrected Akaike Information Criterion, k: multiplier for the penalty term for complexity, LL: Log likelihood.

Table S6. BIC model selection results based on MLPE (Maximum-likelihood population-effects) models from the unbounded (UB) region

	BIC	Delta BIC	BICWt	Cum.Wt	LL
Host genetic resistance	-114.22	0	0.52	0.52	63.1
Roads	-112.47	1.75	0.21	0.73	62.23
EVI	-110.71	3.5	0.09	0.82	61.35
Elevation	-110.59	3.62	0.08	0.9	61.29
Temperature	-109.14	5.08	0.04	0.94	60.56
Streams	-108.56	5.66	0.03	0.98	60.27
Land cover	-107.32	6.89	0.02	0.99	59.65
Canopy cover	-104.4	9.82	0	1	58.19
Spatial proximity	-104.38	9.84	0	1	58.18
Host genetic distance	-101.21	13.01	0	1	56.59

BIC: Bayesian Information Criterion, Delta BIC: Difference in BIC scores, LL: Log likelihood.

Table S7. BIC model selection results based on MLPE (Maximum-likelihood population-effects) models from the wildland urban interface (WUI) region.

	BIC	Delta BIC	BICWt	Cum.Wt	LL
Roads	-201.67	0	0.24	0.24	106.93
Host genetic distance	-201.48	0.19	0.22	0.46	106.83
Topography	-201.07	0.61	0.18	0.63	106.62
Spatial proximity	-200.63	1.04	0.14	0.78	106.41
Streams	-199.79	1.89	0.09	0.87	105.98
Landcover	-198.77	2.9	0.06	0.93	105.48
Canopy cover	-198.62	3.06	0.05	0.98	105.4
Host genetic resistance	-196.7	4.97	0.02	1	104.44
Impervious surface	-192.06	9.62	0	1	102.12
Roads	-201.67	0	0.24	0.24	106.93

BIC: Bayesian Information Criterion, Delta BIC: Difference in BIC scores, LL: Log likelihood.

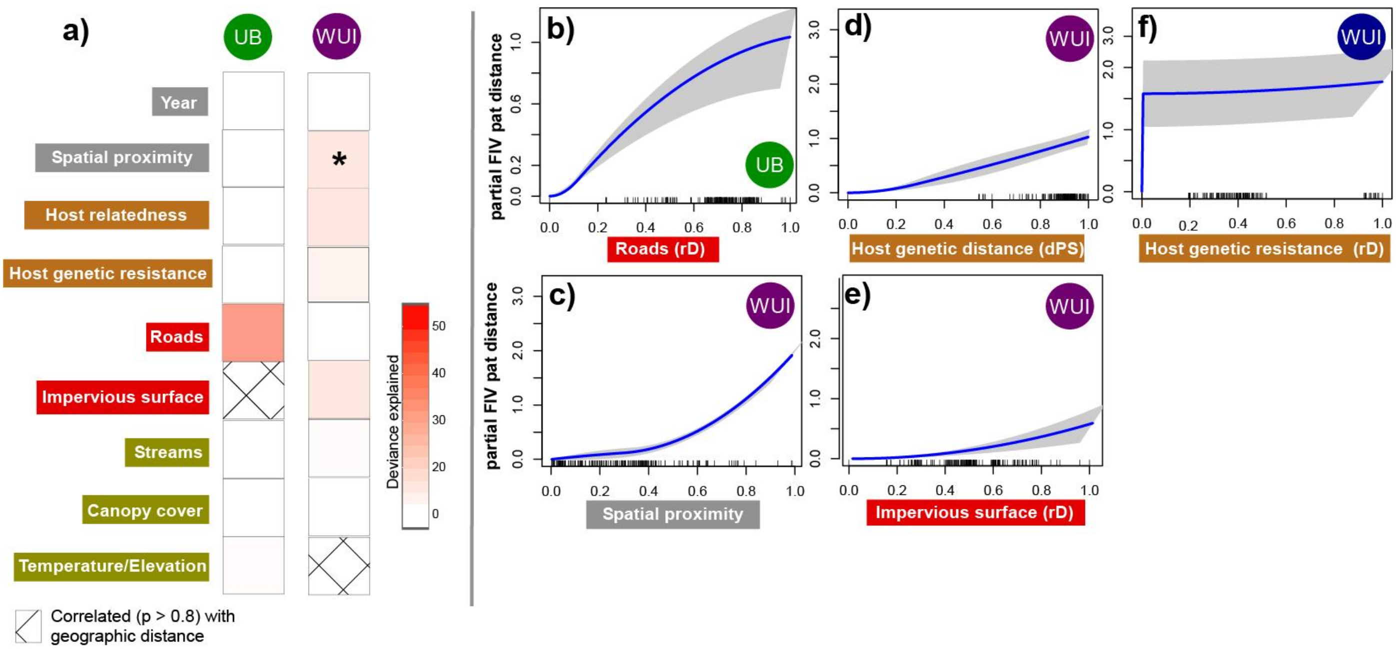

Fig. S5. Results from the GDM analyses of FIV_{pco} spread including host genetic resistance instead of host-genetic optimised resistance. a) Heat map showing the deviance explained by each predictor in the unbounded (UB) and wildland-urban interface (WUI) *: *p* value 0.01-0.05. Predictor labels in grey boxes = spatiotemporal predictors, predictors in orange = host genetic measures, predictors in red = associated with anthropogenic development, predictors in olive green = natural landscape features. The right panel shows partial effect plots (all other variables held at the mean) of the GDM-fitted I-splines showing the relationship between partial FIV_{pco} patristic distance for the variables selected in the UB final model (b, see *Methods*) and WUI (c-f) in order of importance. Pat distance = patristic distance. Lines across the x-axis represent a rug plot showing the distribution of the data and the grey surrounding the blue line (the fit) indicates 95% confidence intervals. rD: Resistance distance. dps: Proportion of shared alleles. See Tables S6/S7 for corresponding MLPE results.

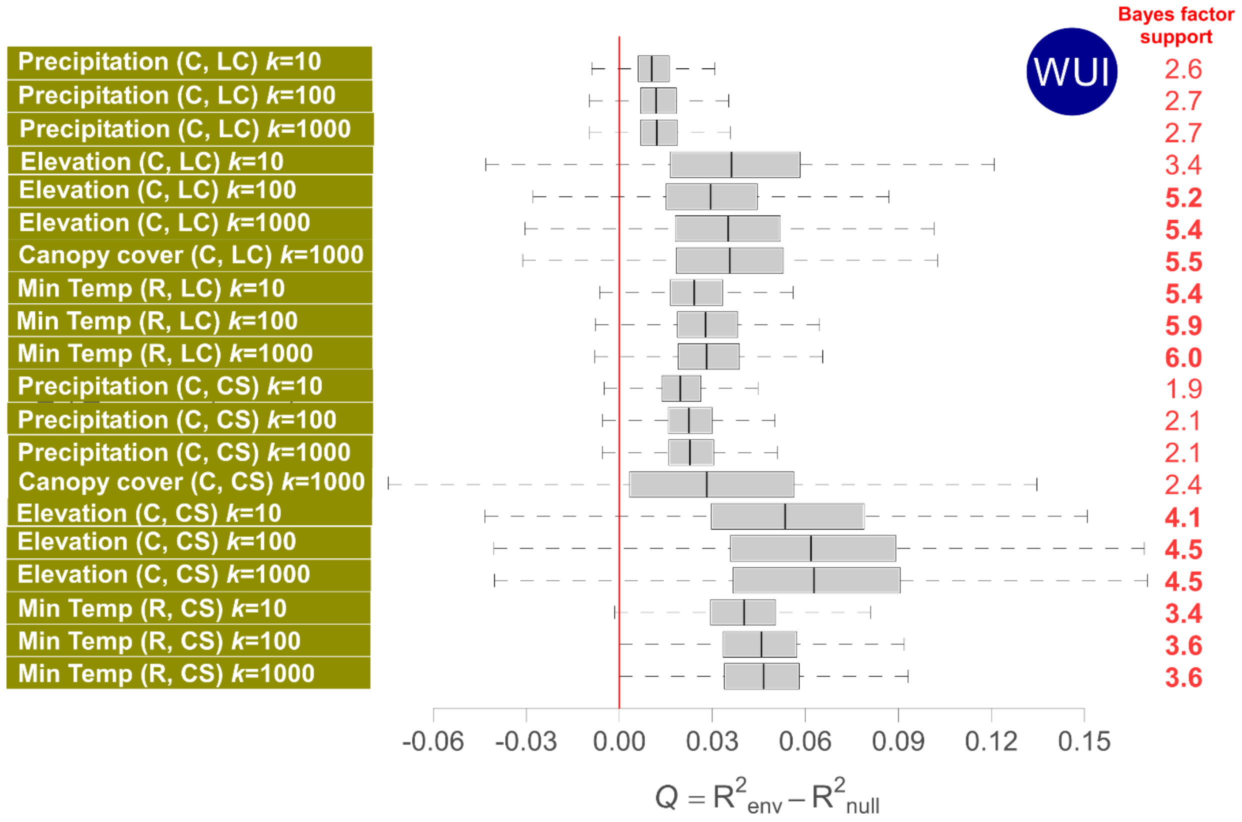

Fig. S6. Analysis of the impact of environmental factors on the dispersal velocity of viral lineages. The figure reports the estimated *Q* distributions and associated approximated Bayes factor (BF) support for the environmental factors associated with at least 90% of positive *Q* values. Here, we report results for the WUI population (CO only) as there were no factors with both positive *Q* distributions [p(*Q*>0) > 0.9)] for the unbounded population. “C” and “R” indicate if the considered environmental variable was considered as a conductance (“C”) or resistance factor (“R”), and *k* is the rescaling parameter used to transform the initial raster. “LC” and “CS” indicate if the environmental distances were computed using the least-cost (“LC”) or Circuitscape (“CS”) path model. Following the scale of interpretation of BF values defined by (17), BF values higher than 3 can be considered as “positive” support and values higher than 20 can be considered “strong” support. Approximated BF values higher than 3 are highlighted in bold.

Text S1.

Sequencing Protocol

Master mix PCR reactions for round 1 - 5 µl Kapa Hotstart hifi polymerase (Kapa biosystems, USA), 2 µl H_2O, 1 µl of both the forward and reverse primer (see below) and 2 µl of DNA., and in round 2 - 10 µl Kapa Hotstart hifi polymerase (Kapa biosystems, USA), 6 µl H_2O, 1 µl of both the forward and reverse primer (see below) and 2 µl of R1 PCR product. The PCR cycling conditions were run according to manufacturer's specifications with an annealing temperature at 60 ºC with reactions run on a C1000 Touch Thermal Cycler (BioRad). Primer details can be found in Table S2 below. PCR products were run on a 0.7 % agarose gel using gel electrophoresis and bands of the correct size excised from the gel. Excised DNA fragments were purified using a MEGAquick-spin™ Total Fragment DNA Purification Kit (iNtRON Biotechnology, Korea). These purified fragments were ligated into pJET 1.2 blunt vector using the CloneJET PCR Cloning Kit (Thermo Fisher Scientific) and cloned using heat shock method with XL1-Blue *E. coli* competent cells (Agilent). Plasmids containing fragments of interest using DNA-spin Plasmid Purification Kit (iNtRON Biotechnology, Korea) and Sanger sequenced using primer walking at Quintarabio (San Francisco, CA).

Table S8. List of primers used to sequence FIV_{pco}. The majority of sequences were obtained using primers starting with PLVB_[pol/env]. For several poorly amplifying pol sequences we developed an additional round two primer PLVB_PolNewF R2. FIV_{pco} subtype WY *env* was amplified using PLVBWY primers.

Name	Gene	Primer sequence
Round 1 PLVB-PolF R1	pol	GAATATGTTSGCKCAAGCYTTACAAC
PLVB_PolR R1	pol	GTGRAAGGACCAAAGAATTCCTTCTAC
Round 2 PLVB-PolF R2	pol	GAATATGTTSGCKCAAGCYTTACAAC
PLVB_PolR R2	pol	CATRTTTCTTTTACTACTCTGYACYCTG
PLVB_PolNewF R2	pol	GCACAAGCGTAACAACAGGT

Table S9. Marginal-likelihood estimates for the candidate models inferred using path-sampling (PS), stepping-stone sampling (SS) for $FIV_{pco}CO$. The model in italics was the model used for subsequent analysis.

Model #	Relaxed-clock	Tree coalescent	Continuous trait model	SS	PS
1	Strict Clock	Constant Size	Brownian	-7460.8	-7460.8
2	UCLN	Constant Size	Brownian	-7449.3	-7449.0
	UCLN	GMRF Bayesian Skyride	Brownian	-7451.9	-7451.3
3	UCLN	Exponential	Brownian	-7446.2	-7446.2
4	UCLN	Exponential	Lognormal RRW	-7413.7	-7413.8
	UCLN	*Exponential*	*Cauchy RRW*	*-7383.5*	*-7383.6*

UCLN: Uncorrelated relaxed clock with a log normal distribution.

Supplementary References

1. Trumbo D, et al. Urbanization impacts apex predator gene flow but not genetic diversity across an urban-rural divide. *Mol Ecol*.

2. Peterman WE (2018) ResistanceGA: An R package for the optimization of resistance surfaces using genetic algorithms. *Methods Ecol Evol* 9(6):1638–1647.

3. Wright S (1942) Statistical genetics and evolution. *Bull Amer Math Soc* 48(4):223–246.

4. Crooks KR (2002) Relative sensitivities of mammalian carnivores to habitat fragmentation. *Conserv Biol* 16(2):488–502.

5. Lewis JS, et al. (2015) The effects of urbanization on population density, occupancy, and detection probability of wild felids. *Ecol Appl* 25(7):1880–1895.

6. Riley SPD, et al. (2006) A southern California freeway is a physical and social barrier to gene flow in carnivores. *Mol Ecol* 15(7):1733–1741.

7. Ernest HB, Vickers TW, Morrison SA, Buchalski MR, Boyce WM (2014) Fractured genetic connectivity threatens a southern California puma (*Puma concolor*) population. *PLoS One* 9(10):e107985.

8. Maletzke B, et al. (2017) Cougar response to a gradient of human development. *Ecosphere* 8(7):e01828.

9. Newby JR, et al. (2013) Human-caused mortality influences spatial population dynamics: Pumas in landscapes with varying mortality risks. *Biol Conserv* 159:230–239.

10. Dickson BG, Jennes JS, Beier P (2005) Influence of vegetation, topography, and roads on cougar movement in southern California. *J Wildl Manage* 69(1):264–276.

11. Hilty JA, Merenlender AM (2004) Use of riparian corridors and vineyards by mammalian predators in northern California. *Conserv Biol* 18(1):126–135.

12. Logan KA, Sweanor LL (2001) *Desert puma : evolutionary ecology and conservation of an enduring carnivore* (Island Press) Available at: https://books.google.com.au/books/about/Desert_Puma.html?id=04joHfHvpsEC&redir_esc=y [Accessed October 24, 2018].

13. Blecha KA, Boone RB, Alldredge MW (2018) Hunger mediates apex predator’s risk avoidance response in wildland-urban interface. *J Anim Ecol* 87(3):609–622.

14. Sweanor LL, Logan KA, Hornocker MG (2000) Cougar dispersal patterns, metapopulation dynamics, and conservation. *Conserv Biol* 14(3):798–808.

15. Hornocker MG, Negri S (2010) *Cougar : ecology and conservation* (University of Chicago Press).

16. McRae BH, Beir P, Dewald LE, Huyhn LY, Keim P (2005) Habitat barriers limit gene flow and illuminate historical events in a wide-ranging carnivore, the American puma. *Mol Ecol* 14(7):1965–1977.

17. Kass RE, Raftery AE (1995) Bayes Factors. *J Am Stat Assoc* 90(430):773–795.
