# Supplementary material for: Host relatedness and landscape connectivity shape pathogen spread in the puma, a large secretive carnivore
Source: Commun Biol. 2021 Jan 4;4:12. doi: 10.1038/s42003-020-01548-2 (PMC7782801; doi:10.1038/s42003-020-01548-2)
Supplement: Supplementary file 3 — Description of Supplementary Files [file 42003_2020_1548_MOESM3_ESM.pdf]

Description of Additional Supplementary Files

File name: Supplementary Data 1

Description: FIVCO1 phylogeographic data

File name: Supplementary Data 2

Description: FIVCO2 phylogeographic data

File name: Supplementary Data 3

Description: FIVCO3 phylogeographic data

File name: Supplementary Data 4

Description: FIVWY phylogeographic data
